# ST-Elevation Myocardial Infarction in a Patient with Anomalous Left Anterior Descending Artery and Absent Left Circumflex

**DOI:** 10.7759/cureus.3311

**Published:** 2018-09-15

**Authors:** Zachary T Luebbering, Obai Abdullah, Abdallah M Mansour, Bhaskar Bhardwaj, Kul Aggarwal

**Affiliations:** 1 Department of Internal Medicine, University of Missouri – Columbia, Columbia, USA; 2 Department of Internal Medicine, Division of Cardiology, University of Missouri – Columbia, Columbia, USA

**Keywords:** coronary artery anomalies, absent left circumflex, non-dominant left anterior descending artery

## Abstract

The left anterior descending artery originating from the right coronary sinus is very unusual. An absent left circumflex is very rare with a few reported cases in the literature. We report a combination of a non-dominant left anterior descending artery arising from the right coronary cusp and an absent left circumflex artery in a 60-year-old male presenting with ST-segment elevation myocardial infarction. The patient was managed by percutaneous intervention and recovered well. This case demonstrates an extremely rare combination of coronary anomalies.

## Introduction

Approximately 99% of the populations’ coronary anatomy consists of a right coronary artery (RCA) originating from the right aortic cusp and a left main coronary artery (LMCA) arising from the left aortic cusp that gives rise to the left anterior descending artery (LAD) and left circumflex (LCX). This leaves 1% of the population with anomalous coronary arteries (CAA) of varying clinical significance [1].

Many of the CAA are benign but can rarely be associated with sudden cardiac death as in LMCA originating the right sinus of Valsalva [2]. We are reporting an extremely rare CAA where the LAD originated from the right coronary cusp along with a super dominant RCA that supplied the area of a missing LCX in a patient with ST-segment elevation myocardial infarction.

## Case presentation

**Figure 1:**
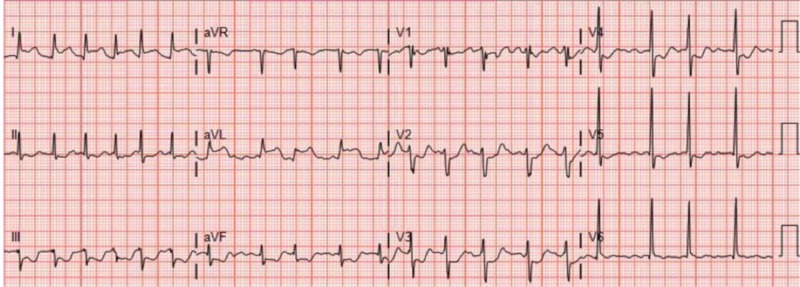
Patient’s electrocardiogram (EKG) on presentation. High lateral ST-segment elevation in leads I and aVL with reciprocal ST depression in lateral and inferior leads.

A 60-year-old male with a history of heart failure with reduced ejection fraction (HFrEF) secondary to severe mitral regurgitation (MR), paroxysmal atrial fibrillation and hypertension presented to the emergency department with typical anginal chest discomfort associated with shortness of breath, diaphoresis, nausea, and vomiting. He denied any alcohol or tobacco use. He was not taking any medications. He was adopted, thus family history was unknown. The patent was afebrile and his heart rate was 91 beats per minute (BPM) with a blood pressure of 107/81 mmHg. His oxygen saturation was 91% on 4 L/min via nasal cannula. On physical exam, he was in respiratory distress, had tachypnea with a rate of 30 breath per minute, was noted to use accessory muscles, the jugular vein was distended but had no peripheral edema. On auscultation of the chest, he had diffuse bilateral crackles. The cardiac exam revealed irregular rhythm with MR murmur. Electrocardiogram (EKG) on presentation demonstrated high lateral ST-segment elevation in leads I and aVL with reciprocal ST depression in anterolateral and inferior leads (Figure 1). An echocardiogram was obtained and revealed an enlarged left atrium, global hypokinesis with mild mitral regurgitation and an ejection fraction of 25–30%.

**Figure 2:**
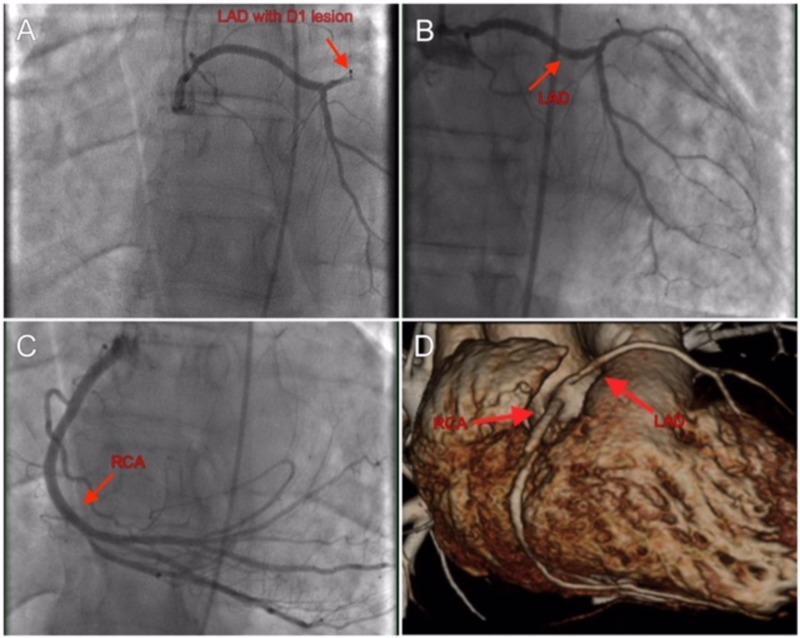
Angiographic findings and cardiac computed tomography (CT) imaging of the anomalous vessels. (A) Left anterior descending artery with 100% D1 lesion. (B) Culprit vessel post intervention. (C) Super-dominant right coronary artery. (D) Cardiac CT showing origin of RCA (left) and LAD (right) from right sinus of Valsalva. RCA: Right coronary artery; LAD: Left anterior descending artery.

He emergently underwent a coronary angiogram. The operator was not able to engage the left coronary system for which an aortic root angiography was performed which failed to reveal any coronary artery take off from the left cusp. Engaging the right coronary system demonstrated an anomalous LAD originating from the right cusp and an absent LCX (Figure 2A). The LCX territory was supplied by a large RCA (Figure 2C). The culprit lesion was a 100% first diagonal (D1) occlusion with grade 0 TIMI flow (Figure 2A). Percutaneous intervention was performed with a drug-eluting stent achieving grade 3 TIMI post-intervention (Figure 2B). To better visualize his coronary anatomy, cardiac computed tomography (CT) was performed (Figure 2D). This showed a rudimentary LAD originating from the right coronary cusp that coursed anterior to the pulmonary artery and bifurcated into two branches. The RCA was dominant and with large caliber arising from the right cusp and at the crux of the heart. It had two branches and continued posterolaterally as a large posterior lateral branch.

Post-intervention, the patient reports resolution of dyspnea and chest pain, was able to ambulate with no difficulties and his oxygen saturation improved throughout his hospital stay and was discharged home with no supplemental oxygen needed. The patient was traveling upon presentation and thus returned home to follow with his primary care physician.

## Discussion

The LAD originating from the right coronary sinus is a very unusual CAA with a frequency of 0.03% [1]. An absent LCX is also very rare CAA with only a few reported cases in the literature and an incidence of 0.003% to 0.067% [1]. To our knowledge, this is one of the very few reports of the combination of these two rare anomalies. The previously published reports described patients who underwent angiography for exertional chest discomfort and were found to have either high-grade stenosis in a dominant RCA [3] or no significant coronary stenosis [4]. We have also found few reports of congenitally absent LCX and super-dominant RCA but with normal LAD origin which might carry similar clinical significance and further emphasize on the importance of precise morphological and functional evaluation of the CAA in selecting the best treatment modality and better prognosis [5-8].

The clinical implications of CAA are variable depending on the type of anomalous artery and range from ischemia presenting at the beginning of life to misdiagnosis secondary to unfamiliar anatomy on angiography [9]. Coronary arteries arising from the opposite sinus (ACAOS) are the second leading cause of sudden cardiac death (SCD) in young athletes in the United States [10]. Surgical intervention may be required for this anomaly [11]. In one-third of young athletes who had sudden death related to ACAOS, prior symptoms of either chest pain or syncope were present [12]. Many of these athletes underwent electrocardiogram (ECG), stress ECG and echocardiogram which were all found to be normal [13]. Most CAAs are found incidentally during angiography [9]. Angiographically, CAA can mimic atherosclerotic coronary ectasia, coronary lesions associated with aneurysms and fistulas following coronary artery surgery [9]. When found incidentally, clinical significance may be determined with invasive or noninvasive pharmacological stress test [9]. Multi-detector cardiac computed tomography (MDCT) has proven to be valuable at determining CAA. Graidis et al. found CAA in 2.33% of patients undergoing MDCT for chest pain, angina equivalents or multiple cardiovascular disease risk factors [13]. Cardiac magnetic resonance imaging (MRI) can also be considered to better delineate anatomical variations and plan for intervention when ionizing radiation and contrast exposure need to be avoided [14]. The type of anomaly described in our case is associated with an increased risk of SCD [2] and is particularly dangerous when, after arising from the right cusp, the LMCA travels between the pulmonary and aortic arteries [15] which puts it through an unusual course with an acute angle which plays a role in increasing the risk of cardiac events [2]. When ACAOS is discovered later in life, the risk of SCD is negligible [1]. Once anatomy is defined, determination of benefit from surgical intervention needs to be made. Current indications for intervention for CAA are mentioned in 2008 American College of Cardiology (ACC) guidelines which recommend intervention for left anomalous coronary artery arising from right sinus of Valsalva if it courses between the aorta and the pulmonary artery, if there is evidence of ischemia from an intramural great vessel course such as when the LMCA or RCA arises from the opposite sinus, and if the RCA passes between the aorta and pulmonary artery after arising from the left sinus [16]. In the case discussed, surgery was not recommended as the LAD was traveling anteriorly to the pulmonary artery and due to the low risk of SCD in an asymptomatic elderly patient. This case demonstrates an asymptomatic and extremely rare combination of anomalies.

## Conclusions

Definition and management of CAA vary widely depending on the type of lesion and physician's preference. Further research is needed to expand the knowledge of risks and benefits of invasive and noninvasive imaging, screening and intervention for CAA.
